# Evaluating the effects of bempedoic acid on lipid profiles and cardiovascular risk: An umbrella review of meta-analyses

**DOI:** 10.5339/qmj.2025.51

**Published:** 2025-07-05

**Authors:** Ashik Ali, Sameer Bhimani, Vikash Kumar Karmani, Rubaid Azhar Dhillon, Shahzeb Saeed, Arman Amir, Palak Patel, Anim Asif, Umair Abrar Baig, Sheena Shamoon, Aatkah Naseer, Owais Ali

**Affiliations:** 1 Department of Internal Medicine, SRM Medicak College and Research Center, Chennai, India; 2 Department of Internal Medicine, The Wright Center for Graduate Medical Education, Scranton, PA, USA; 3 Department of Internal Medicine, Jinnah Sindh Medical University, Karachi, Pakistan; 4 Department of Internal Medicine, Riphah International University, Rawalpindi, Pakistan; 5 Department of Internal Medicine, Charleston Area Medical Center, Charleston, WV, USA; 6 Department of Internal Medicine, Dow University of Health Sciences, Karachi, Pakistan; 7 Department of Internal Medicine, New York Medical College at Saint Michael’s Medical Center, Newark, NJ, USA; 8 Department of Internal Medicine, Harlem Hospital Center, New York, NY, USA; 9 Department of Cardiology, Orthopedic Medical and Institute, Karachi, Pakistan; 10 Department of Internal Medicine, Rawalpindi Medical University, Rawalpindi, Pakistan; 11 Department of Cardiology, National Institute of Cardiovascular Disorder, Karachi, Pakistan; 12 Department of Internal Medicine, Jinnah Sindh Medical University, Karachi, Pakistan *Email: Arman.maredia1@gmail.com

**Keywords:** Bempedoic acid, major adverse cardiovascular events, lipid profile

## Abstract

**Background:**

This umbrella review aims to synthesize evidence from previously conducted meta-analyses and review articles to assess the effects of bempedoic acid on lipid profile and cardiovascular events.

**Methods:**

While adhering to the Preferred Reporting Items for Overviews of Reviews guidelines, PubMed, Google Scholar, Web of Science, and Scopus were searched from the database inception to June 2024 to identify relevant articles. The outcomes were total cholesterol, low-density lipoprotein cholesterol (LDL-C), high-density lipoprotein cholesterol (HDL-C), non-HDL cholesterol, triglyceride (TAG), apolipoprotein B (APOB), high-sensitivity CRP (hs-CRP), major cardiovascular events (MACE), cardiovascular mortality, and myocardial infarction (MI). A corrected covered area (CCA) assessment was performed to determine overlap among reviews. Each included review was assessed for its quality and rigor via the AMSTAR-2 tool.

**Results:**

From 18,297 articles identified during the literature search, 18 meta-analyses were included. A significant overlap was noted across studies with a corrected cover area of 44.4%. Bempedoic acid’s effects on cardiovascular outcomes and lipid levels have been extensively studied. For cardiovascular mortality, the evidence is mixed: Goyal et al.^21^ reported a risk ratio (RR) of 0.81 (95% CI 0.61–1.08) suggesting a potential benefit, while other studies, such as De Filippo et al.^26^ and Zhang et al.^24^, indicate no significant association. In terms of MACE, 11 reviews show a consistent trend toward reduced risk, with RRs between 0.75 and 0.88. Bempedoic acid also appears to significantly reduce the risk of MI, with RRs and odds ratios (ORs) around 0.76. Evidence on unstable angina suggests a lower risk, although some studies do not reach statistical significance. For coronary revascularization, the data show a reduced risk, with RRs ranging from 0.74 to 0.82. Studies on coronary non-revascularization also indicate a significant risk reduction with RRs and ORs of 0.41. Regarding lipid levels, bempedoic acid consistently reduces LDL cholesterol (mean differences [MDs] from −17.5% to −33.91%), total cholesterol (MDs from −12.69% to −34.41%), and non-HDL cholesterol (MDs from −12.3% to −23.27%). The effects on HDL cholesterol are less consistent (MDs from −1.29% to −5.18%), and triglyceride levels show variable results (MDs from −8.35% to +5.23%).

**Conclusion:**

Our findings show that bempedoic acid significantly reduces the risk of MACE, nonfatal MI, coronary and noncoronary revascularization, and hospitalizations for unstable angina. While results on cardiovascular mortality are mixed, suggesting a need for further study, bempedoic acid proves to be an effective treatment for improving lipid profiles and reducing cardiovascular events, especially in patients who cannot tolerate statins. It presents a valuable option for cardiovascular risk management, potentially enhancing patient outcomes and quality of life. Further research is needed to assess its long-term benefits and broader applicability.

## INTRODUCTION

Atherosclerotic cardiovascular disease (ASCVD) remains the leading cause of mortality worldwide, with approximately 17.6 million deaths annually.^1^ Thus, the prevention of ASCVD takes precedence. Reducing low-density lipoprotein cholesterol (LDL-C) levels is a key approach to preventing ASCVD,^2^ for which statins are highly effective and are the foundation of lipid-lowering treatments recommended by the American College of Cardiology/American Heart Association and the European Society of Cardiology/European Atherosclerosis Society guidelines as the primary choice for managing hyperlipidemia.^3,4^ However, the current guidelines are largely based on clinical trials conducted during the early stages of statin development.^5,6^ Despite strong evidence for the effectiveness and safety of statins, intolerance or perceived intolerance to these drugs is frequently encountered in clinical practice.^7^ This often leads to poor adherence to statin therapy, which is associated with a higher risk of major adverse cardiovascular events.^8,9^ Therefore, there is a need for additional lipid-lowering agents that can be used either as monotherapy or, more effectively, in combination therapy to manage cardiovascular disease (CVD) risk.^10^ Advancements in lipid-lowering treatments have been achieved with the use of ezetimibe (including fixed-dose combinations with statins), PCSK9 monoclonal antibodies (such as alirocumab and evolocumab), and small interfering RNA therapies like inclisiran.^11,12^

More recently, bempedoic acid has been turning heads with its effective lipid-lowering effects. Bempedoic acid works by inhibiting the enzyme adenosine triphosphate-citrate lyase, which inhibits the mevalonate pathway of cholesterol synthesis.^13^ The drug’s activation in the liver and extensive first-pass metabolism lead to the limited exposure of the active compound in the systemic circulation, which accounts for its good tolerability and minimal adverse effects.^14,15^ The recent CLEAR Outcomes trial (Cholesterol Lowering via Bempedoic Acid, an ATP Citrate Lyase-Inhibiting Regimen) evaluated the use of bempedoic acid in high-risk primary and secondary prevention patients who could not or would not take statin therapy due to intolerance.^16^ This review aims to examine the safety and efficacy of bempedoic acid, review the cardiovascular outcomes trial data, and offer considerations for its application in current clinical practice.

## METHODOLOGY

This study is an umbrella review, which is a systematic collection and analysis of previously published systematic reviews and meta-analyses on the topic. The Preferred Reporting Items for Overviews of Reviews (PRIOR) were followed to conduct this review.^17^ For clarity, throughout the paper, we shall refer to the existing systematic reviews and meta-analyses as “reviews,” while the studies included in each systematic review and meta-analysis shall be referred to as “studies.”

## DATA SOURCES AND LITERATURE SEARCH

Data sources and literature search to find relevant reviews for inclusion in this umbrella review, two authors (AA and RD) independently conducted systematic searches of electronic databases like PubMed, Google Scholar, Scopus, and Web of Science from their inception until June 2024 and with a third investigator (VK) resolving any disagreements that arose during the selection process. No language or geographical restrictions were applied. The search strategy included relevant keywords such as “bempedoic acid” and “hypercholesterolemia OR lipid-lowering therapy” and “cardiovascular events,” with the complete strategy for each database detailed in Table 1. The search results from all databases were combined, and duplicates were removed. After an initial screening of titles and abstracts, full texts of the selected review articles were obtained and further assessed for adherence to the inclusion and exclusion criteria.

## INCLUSION AND EXCLUSION CRITERIA

1. Eligible studies for this umbrella review include systematic reviews and meta-analyses.
2. Patients aged >18 years diagnosed with hypercholesterolemia, CVD, or at high risk for CVD.
3. Statin-intolerant patients or those on statins with ASCVD, familial hypercholesterolemia, or multiple cardiovascular risk factors.
4. Treatment with 180 mg bempedoic acid alone or in combination therapy (e.g., with ezetimibe). Control groups receiving a placebo or oral ezetimibe 10 mg once daily.
5. Inclusion of patients with or without PRIOR statin therapy.
6. Studies published in English, conducted in humans, and available as full-text articles for review.

We excluded studies that were unable to extract data, including guidelines, review articles, animal studies, case reports, letters, posters, and conference abstracts, as well as other fundamental studies and book chapters OR studies investigating bempedoic acid dosages other than 180 mg and without a comparison between the bempedoic acid and placebo group or the absence of a placebo group.

The outcomes were total cholesterol, LDL-C, high-density lipoprotein cholesterol (HDL-C), non-HDL cholesterol, triglyceride (TAG), apolipoprotein B (APOB), high-sensitivity CRP (hs-CRP), major cardiovascular events (MACE), cardiovascular mortality, and myocardial infarction (MI).

## DATA EXTRACTION

VK, AA, and PP extracted all data independently. Differences are resolved through discussion and consensus. Extract the following data from the final included article: first author and publication year, study type, number of studies included in the review, and total participants.

## ASSESSMENT OF METHODOLOGICAL QUALITY

VK, AA, and SB independently used AMSTAR-2 to evaluate the methodological quality of each meta-analysis. The AMSTAR-2 tool provides a comprehensive critical evaluation tool to evaluate the systematic review of health interventions.^18^ AMSTAR-2 consists of 16 items, 7 of which are key areas. Each review was scored on whether there were methodological flaws in key or non-critical items. The grades are “High,” “Moderate,” “Low,” and “Critically low.” Disagreements were resolved through discussions, although a provision had been made to consult a fourth reviewer if necessary.

## CORRECTED COVERED AREA (CCA) INDEX

When several meta-analyses investigated the same outcome, we conducted a CCA index analysis on the repeated, included literature. The degree of overlap in studies was assessed and calculated via the CCA index method.^19^ CCA within the range 0%–5% expresses a slight overlap, 6%–10% expresses a moderate overlap, 11%–15% expresses a high overlap, and >15% expresses a very high overlap, as shown in Table 4.

## RESULTS

### Literature review

**Figure 1:**
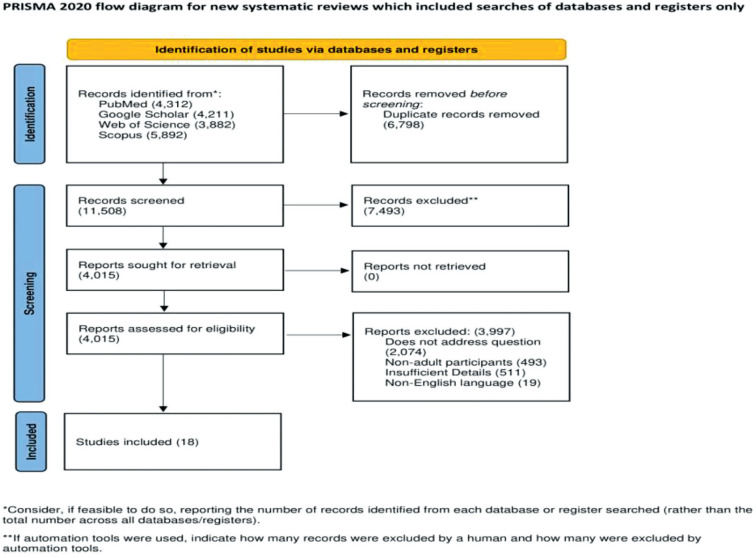
PRISMA study flow chart. PRISMA: preferred reporting items for systematic reviews and meta-analyses.^17^

From 18,297 records identified in the database search, 4,015 were assessed for eligibility. Ultimately, 18 reviews met the inclusion criteria and were included in the umbrella review.^20–37^ Figure 1 outlines the exclusion process at each literature search step. On average, each review incorporated eight studies and a follow-up duration of 4–52 weeks average. The characteristics of these systematic reviews and meta-analyses are detailed in Table 2.

### Quality assessment

Each study was evaluated using the AMSTAR-2 rubric, as shown in Table 3. All included reviews scored 9, indicating high quality. They conducted comprehensive literature searches and used appropriate data synthesis methods. The reviews detailed the characteristics and quality assessments of the included articles. They also considered the scientific quality of the studies in their conclusions and evaluated the potential for publication bias. Additionally, all reviews included a statement declaring no conflicts of interest.

### CCA

The review articles collectively included 18 primary studies. The CCA analysis revealed a substantial overlap of 44.4% among these studies. Table 4 illustrates the extent of overlap observed across the review articles.

## OUTCOMES

### Cardiovascular mortality

Five reviews^21,24,26,28,33^ indicated in Table 5 have explored the relationship between bempedoic acid and cardiovascular mortality using risk ratios (RR) or odds ratios (OR) with corresponding confidence intervals (CI). Goyal et al.^21^ reported an RR of 0.81 (95% CI 0.61–1.08), suggesting a potential but non-significant protective effect. De Filippo et al.^26^ found an OR of 1.04 (95% CI 0.88–1.24), indicating no significant association. Zhang et al.^24^ reported an RR of 1.05 (95% CI 0.89–1.24), suggesting a slight increase in risk without statistical significance. Lin et al.^28^ and Wang et al.^33^ reported wide CIs with ORs of 1.66 (95% CI 0.45–6.04) and 1.65 (95% CI 0.46–5.98), respectively, indicating inconclusive findings.

### Major adverse cardiovascular events

A total of 11 reviews^20,21,23–28,32,33,35^ indicated in Table 5 assessing the risk of MACE and bempedoic acid. Tenorio et al.^20^ reported an RR of 0.86 with a 95% CI of 0.80 to 0.94. Similarly, Goyal et al.^21^ found an RR of 0.81, with a wider CI of 0.61–1.08. Previous studies include Venkatraman et al.^23^ with an RR of 0.88 (CI 0.77–0.99), Zhang et al.^24^ with an RR of 0.86 (CI 0.87–0.94), Uddin et al.^25^ with an RR of 0.87 (CI 0.80–0.94), and De Filippo et al.^26^ and Mutschlechner et al.^27^ reporting OR of 0.86 (CI 0.79–0.95) and 0.84 (CI 0.76–0.96), respectively. Earlier studies by Lin et al.^28^ and Wang et al.^33^ noted ORs of 0.85 (CI 0.61–1.15) and RRs of 0.75 (CI 0.56–0.99), respectively. Khan et al.^35^ and Bhagavathula et al.^32^ also contributed with RRs of 0.82 (CI 0.61–1.11) and 0.34 (CI 0.04–3.17), respectively. These findings collectively suggest a trend toward a lower risk of MACE associated with bempedoic acid.

### MI

Four reviews^21,22,26,33^ indicated in Table 5 have investigated the association between bempedoic acid and the outcome of MI. Goyal et al.^21^ reported an RR of 0.76 (95% CI 0.66–0.88), suggesting a statistically significant reduction in the risk of MI. Li et al.^22^ similarly found an OR of 0.76 (95% CI 0.65–0.90), indicating a significant protective effect against MI. De Filippo et al.^26^ also reported an OR of 0.76 (95% CI 0.64–0.88), further supporting the notion of reduced MI risk with their intervention. In contrast, Wang et al.^33^ showed an RR of 0.54 (95% CI 0.25–1.15), which suggests a non-significant trend toward a lower risk of MI, although the wide CI indicates uncertainty in the effect size.

### Unstable angina

Six reviews^21,24–26,28,33^ indicated in Table 5 have explored the association between bempedoic acid and hospitalization of unstable angina as an outcome. Goyal et al.^21^ reported an RR of 0.67 (95% CI 0.50–0.88), suggesting a lower risk associated with their investigated factor. Similarly, Zhang et al.^24^ found an RR of 0.70 (95% CI 0.55–0.89), indicating a comparable reduction in risk. De Filippo et al.^26^ and Uddin et al.^25^ both reported OR of 0.69 (95% CI 0.54–0.88) and RR of 0.69 (95% CI 0.54–0.88), respectively, further supporting a potentially protective effect against unstable angina. In contrast, Lin et al.^28^ reported an OR of 0.94 (95% CI 0.51–1.74), suggesting no statistically significant association, while Wang et al.^33^ found an RR of 0.84 (95% CI 0.41–1.73), also indicating no strong evidence of a protective effect.

### Coronary revascularization

Six reviews^21,24,25,26,28,33^ indicated in Table 5 have investigated the association between bempedoic acid and coronary revascularization. Goyal et al.^21^ reported an RR of 0.81 (95% CI 0.66–0.99). Similarly, Zhang et al.^24^ found an RR of 0.82 (95% CI 0.73–0.92), while Uddin et al.^25^ reported a comparable RR of 0.82 (95% CI 0.73–0.92). In contrast, De Filippo et al.^26^ presented an OR of 0.81 (95% CI 0.71–0.92) for their study. Lin et al.^28^ provided an OR of 0.82 (95% CI 0.55–1.22), and Wang et al.^33^ reported an RR of 0.74 (95% CI 0.50–1.10). These studies collectively suggest a consistent trend toward reduced risk or odds of coronary revascularization associated with bempedoic acid.

### Coronary non-revascularization

Two reviews^25,28^ indicated in Table 5 have investigated the association between bempedoic acid and coronary revascularization. Uddin et al.^25^ observed a RR of 0.41 (CI 0.18–0.96), indicating a significant association between bempedoic acid and reduced incidence of coronary non-revascularization. Similarly, Lin et al.^28^ found an OR of 0.41 (CI 0.18–0.95) for the same association. Both studies suggest a consistent pattern of bempedoic acid potentially lowering the risk of coronary non-revascularization.

### Low-density lipoprotein cholesterol

Table 6A reported that Goyal et al.^21^ reported an MD of −25.24% (95% CI −30.79% to −19.69%) in LDL cholesterol levels following treatment with bempedoic acid. Li et al.^22^ observed a reduction of −17.5% (95% CI −22.9% to −12.0%), while Tenorio et al.^20^ found a similar decrease with an MD of −20.69% (CI −23.20% to −18.19%). These results are consistent with earlier studies by Venkatraman et al.,^23^ Zhang et al.,^24^ and Uddin et al.,^25^ which also demonstrated significant reductions in LDL cholesterol levels ranging from −19.41% to −33.91%. Across different time frames from Lin et al.^28^ to Minno et al.,^37^ bempedoic acid consistently showed efficacy in lowering LDL cholesterol, with reductions ranging from −16.42% to −26.58%.

### Total cholesterol

Table 6B demonstrated that Goyal et al.^21^ reported an MD of –21.28% (95% CI –30.58 to –11.98), suggesting a substantial reduction. Venkatraman et al.^23^ and De Filippo et al.^26^ also observed significant reductions with MDs of –34.41% (95% CI –42.43 to –26.39) and –16.50% (95% CI –19.21 to –13.79), respectively. Lin et al.^28^ and Dai et al.,^30^ in earlier studies, found MDs of –17.2% (95% CI –22.62 to –11.61) and –12.69% (95% CI –16.31 to –9.06), respectively.

### Non-high-density lipoprotein cholesterol

Table 6C mentioned that Goyal et al.^21^ reported an MD of –23.27 (95% CI –29.80 to –16.73), indicating a substantial decrease in cholesterol levels. Li et al.^22^ found a similar trend with an MD of –12.3% (95% CI –15.3 to –9.20). De Filippo et al.^26^ observed an MD of –20.29 (95% CI –22.56 to –18.01), supporting previous findings. Lin et al.^28^ and Masson et al.^29^ reported MDs of –21.54% (95% CI –28.48 to –14.6) and –15.5% (95% CI –18.1 to –13.0), respectively, indicating significant reductions in cholesterol levels. Dai et al.^30^ and Wang et al.^41^ also demonstrated reductions with MDs of –14.97% (95% CI –19.38 to –10.57) and –21.54 (95% CI –28.48 to –14.6), respectively. Bhagavathula et al.,^32^ Cicero et al.,^36^ and Minno et al.^37^ found MDs of –18.36% (95% CI –24.60 to –12.12), –18.17% (95% CI –21.14 to –15.19), and –19.93% (95% CI –21.55 to –18.31), respectively, further supporting the consistent efficacy of bempedoic acid in lowering total cholesterol levels over recent years.

### HDL-C

Table 6D reported that Goyal et al.^21^ reported an MD of −3.37% (95% CI −3.37 to −3.01), followed by Venkatraman et al.^23^ with an MD of −2.40% (CI −3.09 to −1.71), and Lin et al.^28^ with a decrease of −1.29% (CI −4.19 to 1.61). Dai et al.^30^ and Wang et al.^41^ similarly found reductions in HDL-C (MD = −5.18%, CI −6.19 to −4.16 and MD = −1.29%, CI −4.19 to 1.61, respectively). Conversely, Bhagavathula et al.,^32^ Cicero et al.,^36^ and Minno et al.^37^ reported varied effects, including no significant change or slight increases in HDL-C levels.

### TAG

Table 6E reported that in 2024, Goyal et al.^21^ reported a minimal MD of 0.07% (95% CI −7.86 to −8.01), suggesting a slight reduction in TAG levels. Venkatraman et al.^23^ found a modest MD of −1.22% (95% CI −2.50 to 4.94), indicating inconclusive effects with wide CIs. Lin et al.^28^ observed a larger MD of 5.23% (95% CI −16.45 to 27.01), demonstrating significant variability in outcomes. In contrast, Dai et al.^30^ and Wang et al.^41^ reported conflicting MDs of 0.96% (95% CI −4.08 to 6.01) and 5.23% (95% CI −16.45 to 27.01), respectively. Bhagavathula et al.^32^ noted a notable negative MD of −8.35% (95% CI −16.08 to −0.63), suggesting a potential TAG-lowering effect. Cicero et al.^36^ reported a small negative MD of −1.51% (95% CI −3.75 to 0.74), indicating a minimal impact. Minno et al.^37^ found a positive MD of 3.35% (95% CI −1.78 to 8.49), indicating a moderate increase in TAG levels.

## DISCUSSION

Our findings show that bempedoic acid significantly lowers the risk of MACE, nonfatal MI, and both coronary and noncoronary revascularization, along with reducing hospitalizations for unstable angina. As a novel lipid-modifying agent, bempedoic acid provides a promising alternative for patients with moderate hyperlipidemia who have not met treatment goals despite maximal lipid-lowering therapy or who are intolerant to statins. These benefits underscore the potential of bempedoic acid to address an essential gap in cardiovascular risk management for these patients. Our study found mixed results regarding cardiovascular mortality, with some reviews suggesting a potential protective effect while others showed no significant association. For example, Goyal et al.^21^ reported an RR of 0.81 (95% CI 0.61–1.08), indicating a potential but non-significant reduction in cardiovascular mortality. In contrast, De Filippo et al.^26^ found an OR of 1.04 (95% CI 0.88–1.24), suggesting no significant effect on mortality. High-sensitivity C-reactive protein is a well-recognized indicator for predicting future coronary events.^38^ Four phase 3 clinical trials have shown that bempedoic acid significantly lowers LDL-C and high-sensitivity C-reactive protein levels, indicating potential cardiovascular benefits and anti-inflammatory effects.^38^ In the CLEAR Outcomes trial, after 12 months of treatment, bempedoic acid reduced high-sensitivity C-reactive protein levels by 0.34 mg/L (95% CI −0.42 to −0.29), whereas the placebo group saw a reduction of 0.01 mg/L (95% CI −0.04 to 0.09).^39^ The fixed-dose combination of bempedoic acid and ezetimibe has shown even greater efficacy in reducing LDL-C as well as high-sensitivity C-reactive protein levels. Although the main goal of therapy is to reduce LDL-C, bempedoic acid has also consistently demonstrated a reduction in high-sensitivity C-reactive protein levels, offering cardiovascular benefits. A study by Ridker et al.^40^ used canakinumab as a therapeutic agent in atherosclerotic disease patients, reducing inflammation by lowering high-sensitivity C-reactive protein levels without affecting lipid levels. As a result, patients taking canakinumab had a significantly lower rate of cardiovascular events compared to the placebo group, independent of lipid levels. This study demonstrates that targeting inflammation can lower cardiovascular events in high-risk patients. Bempedoic acid has been shown to effectively lower LDL cholesterol levels, which is a crucial factor in managing cardiovascular risk. In the CLEAR Outcomes trial, the bempedoic acid group had fewer cardiovascular deaths compared to the placebo group, with 37 patients (1.8%) versus 65 patients (3.1%).^39^ Similarly, all-cause mortality was also lower in the bempedoic acid group compared to the placebo group, with 75 patients (3.6%) versus 109 patients (5.2%).^39^ Another study conducted by Gunn et al.^41^ conducted a simulation study to analyze the benefits of bempedoic acid among patients with ASCVD. This study predicted that bempedoic acid reduced the 10-year cardiovascular event risk by 10% compared to the placebo group. These findings align with our observations, reinforcing the efficacy of bempedoic acid in reducing major cardiovascular events in statin-intolerant patients. Our study found that bempedoic acid significantly reduces the risk of nonfatal MI. Goyal et al.^21^ reported an RR of 0.76 (95% CI 0.66–0.88), suggesting a statistically significant reduction in MI risk. Similarly, Li (2024) found an OR of 0.76 (95% CI 0.65–0.90), indicating a strong protective effect against MI. Our review and the CLEAR Outcomes trial both noted significant reductions in individual components of MACE. For instance, in the trial, bempedoic acid was associated with a 31% reduction in MI and a 22% reduction in coronary revascularization procedures compared to placebo.^39^ A meta-analysis conducted by Cordero et al.^42^ of four clinical trials examining the effects of bempedoic acid on patients found significant reductions of MACE by 12%, MI by 24%, and coronary revascularization by 18%, in addition to lower all-cause mortality. In our review, Goyal et al.^21^ reported an RR of 0.81 (95% CI 0.66–0.99) for coronary revascularization, while Zhang (2023) found an RR of 0.82 (95% CI 0.73–0.92), indicating a substantial reduction in the need for these procedures. A meta-analysis conducted by Sayed et al.^43^ also reported that bempedoic acid reduces the risk of major cardiovascular events such as MI, revascularization, and unstable angina hospitalization. These consistent results across various studies underscore the robust cardiovascular benefits of bempedoic acid, particularly in preventing recurrent cardiovascular events. The other alternative treatments available that reduce LDL-C and cardiovascular risk are a combination of ezetimibe, alirocumab, and evolocumab.^43^ The IMPROVE-IT trial conducted on ezetimibe and the FOURIER trial on evolocumab both saw similar reductions in MACE compared to the CLEAR Outcomes trial.^44,45^ Non-significant reductions were reported similar to our analysis of cardiovascular mortality. However, bempedoic acid increased the likelihood of gout, renal impairment, and cholelithiasis, which were not significantly affected by ezetimibe or evolocumab. Interestingly, bempedoic acid also lowered the risk of myalgia, suggesting it could be beneficial for patients who suffer from persistent myalgia after beginning statin treatment.^45^ In summary, while bempedoic acid may not significantly impact cardiovascular mortality, its ability to reduce MACE, MI, and revascularization rates, along with its lipid-lowering effects, makes it a promising alternative for statin-intolerant patients. These benefits underscore the potential of bempedoic acid to fill an essential gap in the management of cardiovascular risk in these patient populations. Furthermore, the ability of bempedoic acid to reduce the need for coronary revascularization procedures can have significant implications for healthcare systems. By potentially decreasing the number of invasive procedures required, bempedoic acid not only improves patient outcomes but also may contribute to reducing healthcare costs associated with complex cardiovascular interventions. Multiple studies, including those by Goyal et al.^21^ and Li (2024), have reported substantial reductions in LDL-C levels with bempedoic acid. These studies found MDs ranging from −17.5% to −25.24%, aligning with the CLEAR Outcomes trial’s 21% reduction.^39^ Bempedoic acid becomes active as bempedoyl-CoA in the liver, inhibiting ATP citrate lyase, which reduces the synthesis of acetyl-CoA, a key precursor for cholesterol.^46^ LDL receptors are upregulated due to decreased cholesterol production in the liver. These receptors remove LDL from the bloodstream into the liver, thereby effectively lowering blood LDL-C levels. Since this process is restricted to the liver, this explains the low myalgia symptoms reported in patients taking bempedoic acid.^46^ The CLEAR Harmony trial included patients with ASCVD with or without heterozygous familial hypercholesterolemia, who were randomized into two groups: bempedoic acid and placebo.^47^ The bempedoic acid group had LDL-C levels reduced by 16.5% from baseline in addition to the 12% reduction in apolipoprotein B, confirming its efficacy and safety as an adjunct to statin therapy regardless of the statin dose. The CLEAR Wisdom trial also reported similar lipid improvement levels in patients taking bempedoic acid at week 12, where total cholesterol was reduced by 10% and apolipoprotein B by 9%.^48^ A phase three trial was conducted by Ballantyne et al.,^49^ analyzing the effectiveness of bempedoic acid combined with ezetimibe. A total of 301 patients were selected, who had multiple cardiovascular risk factors. At 12 weeks, the group receiving the combination of bempedoic acid and ezetimibe reported a 36.2% reduction in LDL-C levels compared to a 17.2% reduction in the group only taking bempedoic acid. Additionally, there was a 35% reduction in high-sensitivity C-reactive protein levels in the group receiving the combination of medications. These studies support our findings that bempedoic acid significantly improves lipid profiles. These improvements are crucial for managing cardiovascular risk in statin-intolerant patients, reinforcing bempedoic acid’s role as a valuable alternative in lipid-lowering therapy. The introduction of new drugs to lower LDL-C is highly beneficial in the fight against ASCVD, the leading cause of death globally. This development is crucial due to the increasing rates of statin intolerance and the need for aggressive LDL-C treatment goals. However, the high cost of drugs such as evolocumab and bempedoic acid limits their utilization, posing a significant challenge. In contrast, more affordable drugs like ezetimibe are more widely used. In terms of side effects, bempedoic acid may be less preferred due to a minor risk of cholelithiasis and gout. In the CLEAR Outcomes trial, gout was observed in 3.2% and cholelithiasis in 2.2% of the group taking bempedoic acid, compared to 2.2% and 1.2% in the placebo group, respectively.^39^ However, patients receiving bempedoic acid reported less myalgia, which could be a significant advantage for those experiencing statin-associated myalgia.

## CONCLUSION

Our findings demonstrate that bempedoic acid significantly reduces the risk of MACE, nonfatal MI, and both coronary and noncoronary revascularization, as well as hospitalizations for unstable angina. The mixed results regarding cardiovascular mortality indicate a need for further research, as bempedoic acid offers a promising and effective treatment option for enhancing lipid profiles and reducing cardiovascular events, particularly in patients who are unable to tolerate statins. Its integration into clinical practice can fill an essential gap in cardiovascular risk management, improving the quality of life and health outcomes for these patients. Further research should continue to explore the long-term benefits and broader applicability of bempedoic acid in diverse patient populations.

## Author’s contribution

SS, VK, AA, UA, PP: the concept and design of the study; SB, SS, AA, AA, AN, OA: data acquisition; VK, AA, AA, OA: performed the DNA extraction and interpreted the results; SS, RD, SB, VK, UA, AA: analyzed the data and drafted the manuscript. All authors critically revised the manuscript, approved the final version to be published, and agreed to be accountable for all aspects of the work.

## Author declaration

The authors declare that this work is original and backed by scientific research and facts.

## Conflicts of interest

The authors declare no conflicts of interest.

## Data availability statement

All data generated or analyzed during this study are included in the published article.

## Ethics approval and consent

Ethical approval and patient consent were unnecessary as this systematic review involves the synthesis of data from previously published studies.

## Figures and Tables

**Table 1.** Detailed search strategy for each database along with retrieved search results.

**Data base**	**Search string**	**Results**
PubMed	(((((bempedoic acid) AND (Hypercholesterolemia)) OR (lipid-lowering therapy)) AND (Cardiovascular Events)) AND (Systematic Review and Meta-analysis)) OR (Meta-analysis)	4,312
Google Scholar	(((((bempedoic acid) AND (Hypercholesterolemia)) OR (lipid-lowering therapy)) AND (Cardiovascular Events)) AND (Systematic Review and Meta-analysis)) OR (Meta-analysis)	4,211
Scopus	(((((bempedoic acid) AND (Hypercholesterolemia)) OR (lipid-lowering therapy)) AND (Cardiovascular Events)) AND (Systematic Review and Meta-analysis)) OR (Meta-analysis)	5,892
Web of Science	(((((bempedoic acid) AND (Hypercholesterolemia)) OR (lipid-lowering therapy)) AND (Cardiovascular Events)) AND (Systematic Review and Meta-analysis)) OR (Meta-analysis)	3,882

**Table 2.** Baseline characteristics.

**Author, year**	**Type of review**	**Total no. of studies included (n)**	**Total participants (n)**	**Follow-up duration (week)**
Goyal et al.^21^	SR and MA	5	18,484	4–52
Li et al.^22^	SR and MA	14	18,469	12–52
Tenorio et al.^20^	SR and MA	12	18, 439	4–52
Venkatraman et al.^23^	SR and MA	17	21,131	4–52
Zhang et al.^24^	SR and MA	4	17,323	24–52
Uddin et al.^25^	SR and MA	7	17,816	4–52
De Filippo et al.^26^	SR and MA	11	18,315	4–52
Mutschlechner et al.^27^	SR and MA	10	18,200	4–52
Lin et al.^28^	SR and MA	6	3,956	4–52
Masson et al.^29^	SR and MA	7	3,892	4–24
Dai et al.^30^	SR and MA	10	4,104	4–52
Wang et al.^31^	SR and MA	5	625	6–12
Bhagavathula et al.^32^	SR and MA	3	388	12
Wang et al.^33^	SR and MA	11	4,391	4–52
Cicero et al.^34^	SR and MA	4	3,369	4–52
Khan et al.^35^	SR and MA	11	4,311	4–52
Cicero et al.^36^	SR and MA	10	3,788	4–52
Minno et al.^37^	SR and MA	7	4,236	4–52

SR: systematic review, MA: meta-analysis.

**Table 3.** Methodological quality assessment of included meta-analyses according to the AMSTAR-2.

**Author, year**	**1**	**2**	**3**	**4**	**5**	**6**	**7**	**8**	**9**	**10**	**11**	**12**	**13**	**14**	**15**	**16**	**AMSTAR-2 grade**
Goyal et al. ^21^	Y	N	Y	Y	N	N	Y	Y	Y	N	Y	Y	N	N	Y	N	High quality
Li et al.^22^	Y	N	Y	Y	N	N	Y	Y	Y	N	Y	Y	N	N	Y	N	High quality
Tenorio et al.^20^	Y	N	Y	Y	N	N	Y	Y	Y	N	Y	Y	N	N	Y	N	High quality
Venkatraman et al.^23^	Y	N	Y	Y	N	N	Y	Y	Y	N	Y	Y	N	N	Y	N	High quality
Zhang et al.^24^	Y	N	Y	Y	N	N	Y	Y	Y	N	Y	Y	N	N	Y	N	High quality
Uddin et al.^25^	Y	N	Y	Y	N	N	Y	Y	Y	N	Y	Y	N	N	Y	N	High quality
De Filippo et al.^26^	Y	N	Y	Y	N	N	Y	Y	Y	N	Y	Y	N	N	Y	N	High quality
Mutschlechner et al.^27^	Y	N	Y	Y	N	N	Y	Y	Y	N	Y	Y	N	N	Y	N	High quality
Lin et al.^28^	Y	N	Y	Y	N	N	Y	Y	Y	N	Y	Y	N	N	Y	N	High quality
Masson et al.^29^	Y	N	Y	Y	N	N	Y	Y	Y	N	Y	Y	N	N	Y	N	High quality
Dai et al.^30^	Y	N	Y	Y	N	N	Y	Y	Y	N	Y	Y	N	N	Y	N	High quality
Wang et al.^31^	Y	N	Y	Y	N	N	Y	Y	Y	N	Y	Y	N	N	Y	N	High quality
Bhagavathula et al.^32^	Y	N	Y	Y	N	N	Y	Y	Y	N	Y	Y	N	N	Y	N	High quality
Wang et al.^33^	Y	N	Y	Y	N	N	Y	Y	Y	N	Y	Y	N	N	Y	N	High quality
Cicero et al.^34^	Y	N	Y	Y	N	N	Y	Y	Y	N	Y	Y	N	N	Y	N	High quality
Khan et al.^35^	Y	N	Y	Y	N	N	Y	Y	Y	N	Y	Y	N	N	Y	N	High quality
Cicero et al.^36^	Y	N	Y	Y	N	N	Y	Y	Y	N	Y	Y	N	N	Y	N	High quality
Minno et al.^37^	Y	N	Y	Y	N	N	Y	Y	Y	N	Y	Y	N	N	Y	N	High quality

Y: yes, N: no.

**Table 4.** CCA.

**Total outcomes**	**No. of reviews**	**No. of included studies**	**CCA statistic (%)**	**Degree of overlapping**
12	18	17	44.4%	Very high overlapped

CCA: corrected cover area.

**Table 5.** Effects of bempedoic acid on cardiovascular outcomes.

**Review**	**Effect estimate (95% CI)**
**Cardiovascular mortality**	
Goyal et al.^21^	RR = 0.81, CI 0.61–1.08
De Filippo et al.^26^	OR = 1.04, CI 0.88–1.24
Zhang et al.^24^	RR = 1.05, CI 0.89–1.24
Lin et al.^28^	OR = 1.66, CI 0.45–6.04
Wang et al.^33^	RR = 1.65, CI 0.46–5.98
**MACE**	
Tenorio et al.^20^	RR = 0.86, CI 0.80–0.94
Goyal et al.^21^	RR = 0.81, CI 0.61–1.08
Venkatraman et al.^23^	RR = 0.88, CI 0.77–0.99
Zhang et al.^24^	RR = 0.86, CI 0.87–0.94
Uddin et al.^25^	RR = 0.87, CI 0.80–0.94
De Filippo et al.^26^	OR = 0.86, CI 0.79–0.95
Mutschlechner et al.^27^	OR = 0.84, CI 0.76–0.96
Lin et al.^28^	OR = 0.85, CI 0.61–1.15
Wang et al.^33^	RR = 0.75, CI 0.56–0.99
Khan et al.^35^	RR = 0.82, CI 0.61–1.11
Bhagavathula et al.^32^	RR = 0.34, CI 0.04–3.17
**MI**	
Goyal et al.^21^	RR = 0.76, CI 0.66–0.88
Li et al.^22^	OR = 0.76, CI 0.65–0.90
De Filippo et al.^26^	OR = 0.76, CI 0.64–0.88
Wang et al.^33^	RR = 0.54, CI 0.25–1.15
**Unstable angina**	
Goyal et al.^21^	RR = 0.67, CI 0.50–0.88
Zhang et al.^24^	RR = 0.70, CI 0.55–0.89
De Filippo et al.^26^	OR = 0.69, CI 0.54–0.88
Uddin et al.^25^	RR = 0.69, CI 0.54–0.88
Lin et al.^28^	OR = 0.94, CI 0.51–1.74
Wang et al.^33^	RR = 0.84, CI 0.41–1.73
**Coronary revascularization**	
Goyal et al.^21^	RR = 0.81, CI 0.66–0.99
Zhang et al.^24^	RR = 0.82, CI 0.73–0.92
De Filippo et al.^26^	OR = 0.81, CI 0.71–0.92
Uddin et al.^25^	RR = 0.82, CI 0.73–0.92
Lin et al.^28^	OR = 0.82, CI 0.55–1.22
Wang et al.^33^	RR = 0.74, CI 0.50–1.10
**Coronary non-revascularization**	
Uddin et al.^25^	RR = 0.41, CI 0.18–0.96
Lin et al.^28^	OR = 0.41, CI 0.18–0.95

RR: risk ratio, OR: odd ratio, CI: confidence interval, MACE: major cardiovascular events.

**Table 6A.** LDL-C.

**Review**	**Effect estimate (95% CI)**
Goyal et al.^21^	MD = −25.24%, CI −30.79 to −19.69
Li et al.^22^	MD = −17.5%, 95% CI −22.9% to −12.0
Tenorio et al.^20^	MD = −20.69%, CI −23.20 to −18.19
Venkatraman et al.^23^	MD = −33.91%, CI −39.66 to −28.17
Zhang et al.^24^	MD = −19.41%, CI − 20.46 to − 18.35
Uddin et al.^25^	MD = −22.38%, CI −25.94 to −18.82
De Filippo et al.^26^	MD = −22.42%, CI −24.02 to −20.82
Lin et al.^28^	MD = –26.58%, CI –35.50 to –17.66
Masson et al.^29^	MD = −20.3%, CI −23.5 to −17.1
Dai et al.^30^	MD = −16.42%, 95% CI, −18.16 to −14.69
Wang et al.^41^	MD = –26.58%, CI –35.50 to –17.66
Bhagavathula et al.^32^	MD = −29.14%, CI −39.52 to −18.76
Wang et al.^33^	MD = −22.91%, CI − 27.35 to − 18.47
Cicero et al.^36^	MD = −22.94%, CI −26.63 to −19.25
Minno et al.^37^	MD = –19.93%, CI –21.55 to –18.31

MD: mean difference, CI: confidence interval.

**Table 6B.** Total cholesterol.

**Review**	**Effect estimate (95% CI)**
Goyal et al.^21^	MD = −21.28%, CI −30.58 to −11.98
Li et al.^22^	MD = −10.9%, CI, −13.3 to −8.5
Venkatraman et al.^23^	MD = −34.41%, CI −42.43 to−26.39
Uddin et al.^25^	MD = −13.86%, CI −15.82 to −11.91
De Filippo et al.^26^	MD = −16.50 CI, −19.21 to −13.79
Lin et al.^28^	MD = –17.2%, CI –22.62 to –11.61
Dai et al.^30^	MD = –12.69%, CI –16.31 to –9.06
Wang et al.^41^	MD = –17.12%, CI –22.62 to –11.61
Bhagavathula et al.^32^	MD = −15.78%, CI −20.84 to −10.72
Cicero et al.^36^	MD = −14.94%, CI −17.31 to −12.57
Minno et al.^37^	MD = –12.43%, CI –13.42 to –11.43

MD: mean difference, CI: confidence interval.

**Table 6C.** Non-HDL cholesterol.

**Review**	**Effect estimate (95% CI)**
Goyal et al.^21^	MD = −23.27, CI −29.80 to −16.73
Li et al.^22^	MD = −12.3%, CI −15.3 to −9.20
De Filippo et al.^26^	MD = −20.29, CI −22.56 to −18.01
Lin et al.^28^	MD = –21.54%, CI –28.48 to –14.6
Masson et al.^29^	MD = −15.5%, CI −18.1 to −13.0
Dai et al.^30^	MD = –14.97%, CI–19.38 to–10.57
Wang et al.^41^	MD = –21.54, CI –28.48 to –14.6
Bhagavathula et al.^32^	MD = −18.36%, CI −24.60 to −12.12
Cicero et al.^36^	MD = −18.17%, CI −21.14 to −15.19
Minno et al.^37^	MD = –19.93%, CI –21.55 to –18.31

MD: mean difference, CI: confidence interval.

**Table 6D.** HDL-C.

**Review**	**Effect estimate (95% CI)**
Goyal et al.^21^	MD = −3.37%, CI −3.37 to −3.01
Venkatraman et al.^23^	MD = −2.40%, CI −3.09 to −1.71
Lin et al.^28^	MD = –1.29%, CI –4.19 to 1.61
Dai et al.^30^	MD = –5.18%, CI –6.19 to –4.16
Wang et al.^41^	MD = –1.29%, CI –4.19 to 1.61
Bhagavathula et al.^32^	MD = 1.63%, CI −4.03 to 7.28
Cicero et al.^36^	MD = −3.21%, CI−6.40 to −0.02
Minno et al.^37^	MD = –7.45%, CI –8.30 to –6.61

MD: mean difference, CI: confidence interval.

**Table 6E.** TAG.

**Review**	**Effect estimate (95% CI)**
Goyal et al.^21^	MD = 0.07%, CI −7.86 to −8.01
Venkatraman et al.^23^	MD = −1.22%, CI −2.50 to 4.94
Lin et al.^28^	MD = 5.23%, CI –16.45 to 27.01
Dai et al.^30^	MD = 0.96%, CI –4.08 to 6.01
Wang et al.^41^	MD = 5.23%, 95% CI –16.45 to 27.01
Bhagavathula et al.^32^	MD = −8.35%, CI −16.08 to −0.63
Cicero et al.^36^	MD = −1.51%, CI −3.75 to 0.74
Minno et al.^37^	MD = 3.35, CI –1.78 to 8.49

MD: mean difference, CI: confidence interval.
